# Coating biomimetic nanoparticles with chimeric antigen receptor T cell-membrane provides high specificity for hepatocellular carcinoma photothermal therapy treatment

**DOI:** 10.7150/thno.40291

**Published:** 2020-01-01

**Authors:** Weijie Ma, Daoming Zhu, Jinghua Li, Xi Chen, Wei Xie, Xiang Jiang, Long Wu, Ganggang Wang, Yusha Xiao, Zhisu Liu, Fubing Wang, Andrew Li, Dan Shao, Wenfei Dong, Wei Liu, Yufeng Yuan

**Affiliations:** 1 Department of Hepatobiliary and Pancreatic Surgery, Zhongnan Hospital of Wuhan University, Wuhan, 430071, China.; 2 Key Laboratory of Artificial Micro- and Nano-Structures of Ministry of Education, School of Physics and Technology, Wuhan University, Wuhan, 430072, China.; 3 Key Laboratory of Bio-Medical Diagnostics, Suzhou Institute of Biomedical Engineering and Technology, Chinese Academy of Sciences, Suzhou, Jiangsu 215163, China.; 4 Department of Laboratory Medicine, Zhongnan Hospital of Wuhan University, Wuhan, 430071, China.; 5 Department of Biomedical Engineering, Johns Hopkins School of Medicine, Baltimore, Maryland 21205, USA.; 6 Department of Biomedical Engineering, Columbia University, New York, NY 10027, USA.

**Keywords:** Nanoparticles, chimeric antigen receptor T cell, cell membrane coating technique, photothermal therapy, hepatocellular carcinoma.

## Abstract

**Rationale**: Hepatocellular carcinoma (HCC) is one of the most prevalent malignancies in the world. Apart from traditional surgical resection, radiotherapy, and chemotherapy, more recent techniques such as nano-photothermal therapy and biotherapy are gradually being adopted for the treatment of HCC. This project intends to combine the advantages of nanoscale drug delivery systems with the targeting ability of CAR-T cells.

**Method**: Based on cell membrane-coated nanoparticles and cell membrane-targeting modifications, a novel nanomaterial was prepared by coating CAR-T cell membranes specifically recognizing GPC3^+^ HCC cells onto mesoporous silica containing IR780 nanoparticles. Subsequently, the physical properties were characterized, and the *in vitro* and *in vivo* targeting abilities of this nanoparticle were verified.

**Results**: CAR-T cells were constructed which could recognize GPC3 expressed on the cell surface of HCC cells. Then the isolated CAR-T cell membrane was successfully coated on the IR780 loaded mesoporous silica materials, as verified by transmission electron microscopy. The superior targeting ability of CAR-T cell membrane coated nanoparticles compared to IR780 loaded mesoporous silica nanoparticles was verified, both *in vitro* and *in vivo*.

**Conclusion**: This new nanomaterial exhibits photothermal antitumor abilities along with enhanced targeting abilities, suggesting a promising strategy for the treatment of HCC.

## Introduction

Hepatocellular carcinoma (HCC) is a solid tumor that is the third most frequent cause of cancer death worldwide 1. Although the treatment of HCC has made some progress in recent years, the incidence of local recurrence and distant metastasis is still high, and the poor prognosis of liver cancer patients has not improved 2. It is impossible to eradicate the residual tumor cells in the circulation with local treatment such as surgery and ablation therapy, and the systemic treatment methods such as chemotherapy are affected by factors such as low sensitivity of liver cancer cells 3, 4.

Recently, intelligent nanoparticles have been developed successfully, with biomimetic cell membrane-based drug delivery systems contributing to progress 5, 6. The main synthetic strategies require tedious chemical synthesis and optimization especially when trying to integrate multiple functional modalities into one single nanoparticle. Besides, compromised biocompatibility, and accelerated blood clearance (ABC) have also been observed for poly(ethylene glycol) (PEG) which was previously believed to be inert 7. Cell membrane-coating of nanoparticles, taking advantage of the excellent biocompatibility and versatile functionality of cell membranes can significantly promote the stability of nanoparticles in physiological conditions, resulting in less leakage of drugs 7, 8. In addition, nanoparticles can exhibit excellent tumor targeting capabilities from membrane coating and subsequent modifications, such as platelet-leukocyte hybrid membrane, erythrocyte membrane and so on 5, 9-17.

Chimeric antigen receptors (CARs) provide T cell populations with defined antigen specificities which target tumors, regardless of the natural T cell receptor. CAR T cells can specifically recognize tumor-associated antigen and eliminate tumor cells by single-chain variable region (ScFv) which derived from monoclonal antibody heavy and light chains and expressed on the cell membrane of CAR-T cells, in a non-major histocompatibility complex-restricted manner. Recent successes in chimeric antigen receptor (CAR) T cell immunotherapy for CD19-positive hematological malignancies have highlighted its potential for treating solid tumors 18-20. However, this remarkable therapeutic effect was not observed, when CAR-T cell treatment was used in solid tumors, due to certain barriers 21-23. Aside from proper screening of tumor antigens, long-term persistence of CAR-T cells and efficient trafficking of CAR-T cells from peripheral blood circulation to tumor sites are also essential.

**Figure 1:**
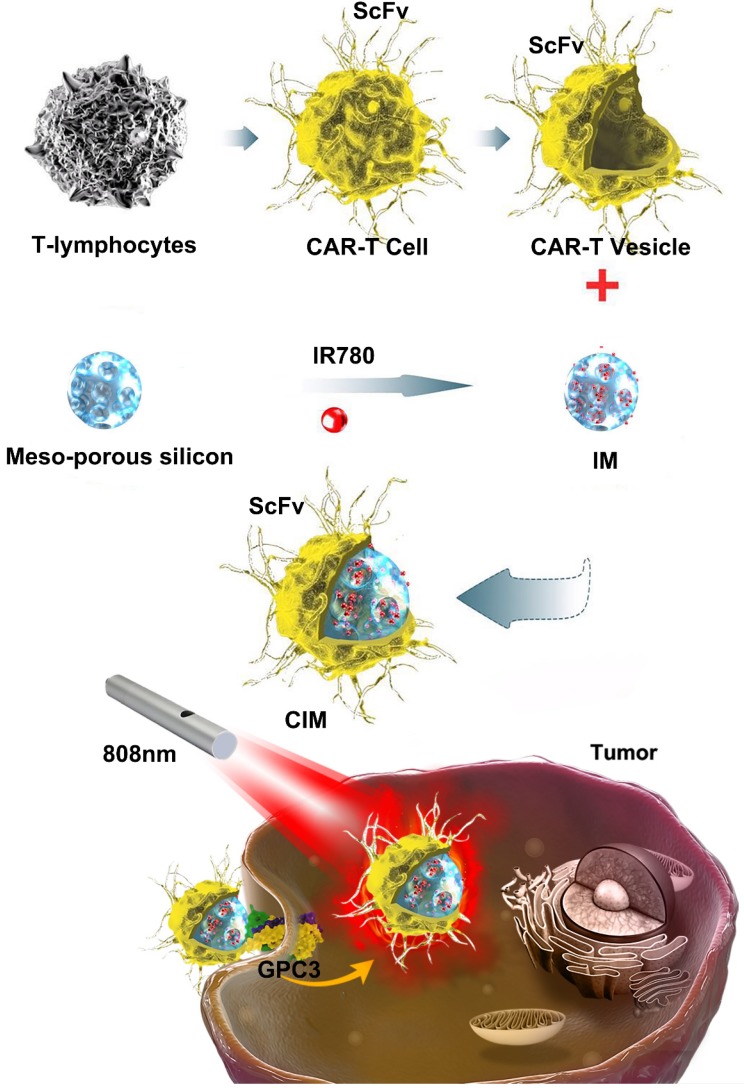
Schematic illustration of chimeric antigen receptor-T (CAR-T) membrane coated biomimetic nanoparticles for highly specific tumor photothermal therapy.

Thus, it is conceivable to combine cell membrane coating nanotechnology with CAR-T therapy to treat solid tumors, due to the high tumor specificity of CAR-T cells and the advantage of cell membrane-camouflaged nanoparticles in drug delivery. In this study, CAR-T membrane-coated nanoparticles were constructed for highly specific therapy for HCC (Figure 1). Glypican-3 (GPC3), a 580-AA heparin sulfate proteoglycan, is expressed in 75 % of HCC samples, but not in healthy liver or other normal tissues 24. Based on this fact, CAR-T cells capable of recognizing GPC3 on the cell membrane of HCC cells have been developed in recent years, which are cytotoxic to GPC3^+^ HCC cells 25, 26. In this study, GPC3 targeting CAR-T cells were first used to prepare CAR-T membranes (CMs). Near-infrared (NIR) dye IR780, a biodegradable photothermal and imaging agent, was then loaded in mesoporous silica nanoparticles (MSNs) to form a biodegradable core. The IR780 dye can produce fluorescence and heat under laser irradiation, and the latter effect can be employed for photothermal therapy 27, 28. Using MSNs for drug delivery is ideal due to their tunable size, good biocompatibility, lack of toxicity, tunable pore sizes (2-20 nm), and enhanced drug-loading capacity 29-31. Taking these factors into consideration, IR780-loaded MSNs (IMs) were used in this paper for photothermal therapy to treat HCC. The IMs were coated with a layer of prefabricated CAR-T membranes using an extrusion method to fabricate tumor specific CAR-T Cell membrane-coated nanoparticles (CIMs). CIMs demonstrated enhanced tumor targeting and anti-tumor capabilities *in vitro* and *vivo*. This targeted nano-system has made significant steps toward further improving nanoparticle functionality in tumor targeted therapy.

## Materials and Methods

### Materials

The MSNs used in this study were obtained from Shanghai Carboxyl Bio-pharmaceutical Technology Co., Ltd. (China). Both IR780 and DAPI were obtained from Wuhan Myhalic Biotechnology Co., Ltd. (China). SDS-polyacrylamide gel and buffer were purchased from Epizyme Scientific (Shanghai, China). All aqueous solutions were prepared using deionized (DI) water. Fluorescein diacetate (FDA), propidium iodide (PI) and cell counting kit-8 (CCK-8) were obtained from Sigma-Aldrich (USA). Other solvents used in this study were purchased from Aladdin-Reagent (Shanghai, China) and Sinopharm Chemical Reagent (Shanghai, China).

### Cell lines and culture

Human HCC cell lines Huh-7 and SK-HEP-1, as well as L02 immortalized human hepatic cell line were purchased from the Cell Bank of Type Culture Collection (Chinese Academy of Sciences, Shanghai, China). All cells were cultured in Dulbecco's modified Eagle's medium (DMEM) with 10 % FBS at 37 °C in a 5 % CO_2_ incubator.

### GPC3-CARs vectors and lentivirus production

The extracellular fragment was composed of GPC3-specific ScFv from GC33 32, linked to the human CD8α hinge and CD28 transmembrane domain. Intracellular signaling domains included the CD28 cytoplasmic domain and CD3ζ molecule. All components were cloned into the second-generation non-self-inactivating SFFV promoter-based lentiviral expression vector pHR. The subsequent production and concentration quantification of lentivirus were completed by Qilu Cell Therapy Technology Co., Ltd. (Shandong, China).

### Isolation, activation, transduction, and culture of T cells

The primary human T cells were enriched with the RosetteSep kit (Stem Cells Technology, Canada) by negative selection of unwanted cells from peripheral blood mononuclear cells (PBMCs) of healthy donors who provided signed consent. Subsequently, the cells were cultured and anti-CD3/anti-CD28 antibodies (ImmunoCult™ Human CD3/CD28 T Cell Activator, Stem Cells Technology, Canada) were added into the T cell culture medium (RPMI 1640, 10 % FBS, 1 % MEM NAA, 50 μM 2-mercaptoethanol, 300 IU/mL rhIL2) to activate primary T cells at day 0 for 24 h. Then, the T cells were transduced with GPC3-CAR lentivirus at a multiplicity of infection (MOI) of 10 with 4 μg/mL polybrene (Santa Cruz Biotechnology, USA). These genetically modified CAR-T cells were used for subsequent assays after proliferation. The expression of GPC3-CAR was analyzed by Fluorescein (FITC) AffiniPure Goat Anti-Mouse IgG, F(ab')2 fragment specific (Jackson ImmunoResearch, USA). The CD4+/ CD8+ T cell population of T cells and CAR-T cells were identified by flow cytometry using, APC anti-Human CD3, PE anti-Human CD4 and FITC anti-Human CD8 antibodies (BD, USA). The CellTrace™ CFSE Cell Proliferation Kit (Invitrogen, USA) was also used to test the cell proliferation disparity between CAR-T cells and T cells.

### Western blot, immunohistochemistry, immunofluorescence and cytotoxicity assay

The expression of GPC3 in Huh7, SK-HEP-1 and L02 cells was detected by western blot, immunohistochemistry and immunofluorescence assays with anti-Glypican 3 antibody (Abcam, USA). The cytotoxicity of GPC3-CAR T cells towards GPC3 overexpressed HCC cells *in vitro* was measured by lactate dehydrogenase (LDH) assay using the CytoTox 96 nonradioactive cytotoxicity kit (Promega, USA). The corrected values were used in the following formula to compute percent cytotoxicity:

Cytotoxicity% = (Experimental - Effector Spontaneous - Target Spontaneous) /(Target Maximum - Target Spontaneous) *100%.

### CAR-T and T membrane isolation

To acquire the cell membranes for nanoparticle coating, T cells and CAR-T cells were washed by PBS twice and then harvested. The cells were suspended in hypotonic lysing buffer consisting of 20 mM Tris-HCl, 10 mM KCl, 2 mM MgCl_2_, and 1 EDTA-free mini protease inhibitor tablet per 10 mL of solution and disrupted using a dounce homogenizer with a tightfitting pestle. The entire solution was subjected to 20 passes before spinning down at 3,200 g for 5 min. The supernatant was saved, while the pellet was resuspended in hypotonic lysing buffer and subjected to another 20 passes and spun down again. The supernatants were pooled and centrifuged at 20,000 g for 30 min, after which the pellet was discarded and the supernatant was centrifuged again at 80,000 g for 1.5 h using an ultra-speed centrifuge (LE-80K, Beckman Coulter, USA). The pellet containing the plasma membrane material was then washed once with 10 mM Tris-HCl and 1 mM EDTA and collected. Then, CAR-T vesicles (CVs) and T cell vesicles (TVs) were obtained by physically extruding the pellet for 11 passes through a 400-nm polycarbonate porous membrane on a mini extruder (Avanti Polar Lipids, USA).

### Preparation of cell membrane coated nanoparticles

To construct IR780-loaded MSNs (IMs), 5 mg of IR780 was dissolved in 1 mL of dimethylsulfoxide (DMSO), and then the solution was added to 4 mL of PBS solution with gentle stirring. The mixture was added dropwise to 10 mL of distilled water containing 10 mg MSNs, and stirred at room temperature overnight to reach equilibrium. The IMs were pelleted by centrifuging at 8000 rpm for 10 min, and washed with distilled water to remove free IR780. CIMs and TIMs (T cell membranes coated IMs) were produced as previously reported 11. Briefly, the collected CVs and TVs were mixed with IMs with sonication. The mixture was subsequently extruded 11 times through a 200 nm polycarbonate porous membrane using an Avanti mini extruder, and then excess vesicles were removed by centrifugation.

### Characterization of cell membrane coated nanoparticles

The particle size and zeta potential of IMs, CAR-T membrane-derived vesicles (CVs), and CIMs were measured by the Malvern Zetasizer ZEN3690 analyzer (Malvern, UK). Transmission electron microscopy (JEM-2010 ES500W, Japan) was used to examine the surface morphologies of the IMs and CIMs, and cell membrane proteins were further examined using sodium dodecyl sulfate-polyacrylamide gel electrophoresis (SDS-PAGE). The protein concentrations of the IMs, T membrane-derived vesicles cell vesicles (TVs), CVs, TIMs and CIMs were quantified with the BCA assay kit (Beyotime Biotechnology, China). After being denatured, 10 μg of each specimen was added into a 10 % SDS-polyacrylamide gel, ran at 80 V for 2 h, and then stained with Coomassie blue (Beyotime Biotechnology, China). Subsequently, the gel was washed by deionized water and imaged. Western blot was also performed to show the successful construction of each membrane coated nanoparticles with AffiniPure Goat Anti-Mouse IgG, F(ab')2 Fragment Specific (Jackson ImmunoResearch, USA).

The concentration of IR780 in CIMs was measured by UV/vis spectrophotometer (Lambda 25, PerkinElmer, USA) based on a standard curve. The drug loading content (DLC) and drug loading efficiency (DLE) of IR780 were calculated as follows: DLC= (weight of feeding IR780 - weight of redundant IR780) / (weight of drug-loaded nanoparticles) × 100 %; DLE = (weight of feeding IR780 - weight of redundant IR780) / (weight of feeding IR780) × 100 % 33. To evaluate the photothermal effects of nanoparticles in PBS solution, IMs, TIMs and CIMs (containing 50 µg/mL IR780) were exposed to 808 nm wavelength laser irradiation (0.6 W/cm^2^) with the illumination direction moving from the top to the bottom of the glass bottle. The negative control was the same volume of PBS with the same laser irradiation. The images of temperature for different nanoparticle dispersions and PBS were captured using an infrared imaging device (ThermaCAM SC3000, FLIR Systems, Inc.) for a total of 5 min. The photothermal temperatures were recorded at different times. The UV-vis absorption spectra of IR780, IMs, MSNs, TIMs and CIMs were measured using a UV-vis spectrophotometer.

### Photothermal Conversion Efficiency

The photothermal conversion efficiency is calculated using Equation:


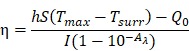


Where h is the heat transfer coefficient, S is the surface of the container; Tmax and Tsurr were the equilibrium temperature and ambient temperature, respectively. Q_0_ is the heat associated with the light absorbance of the solvent, A_λ_ is the absorbance of CIMs nanoparticles at 808 nm, and I is the laser power density. According to the above equation, the η value of CIMs nanoparticles was determined to be about 22.2%.

### Investigation of IR780 release and degradation *in vitro*

The IR780 release from different formulations including the IMS, TIMs and CIMs was determined in PBS at 37 °C. At predetermined time intervals, 100 μL of PBS was taken out from the suspension and replaced with an equal volume of fresh PBS. The released IR780 was obtained by centrifugation, and the R780 concentration was measured using UV-vis spectrometer. A certain amount of CIMs (containing 20μg/mL IR780) were irradiated with NIR irradiation at 0.6 W/cm^2^ for 5 min. Changes in the absorbance and color of the CIMs dispersions were recorded.

### *In vitro* targeting ability and biocompatibility of CIMs

To test the targeting ability of the IR780-loaded formulations, 1×10^5^ Huh-7 cells were cultured in 24-well plates, and treated with PBS, IR780, IMs, TIMs and CIMs (containing 50 μg/mL IR780). After 0.5 h incubation at 37 °C, the treated 24-well plates were washed by PBS, and the cells were stained with DAPI, washed with PBS, and imaged by a confocal laser scanning microscope (CLSM; IX81, Olympus, Japan). The mean fluorescence intensity (MFI) of each group was quantified by a flow cytometer. In addition, the targeting ability of CIMs for different cell lines was evaluated. Briefly, 1×10^5^ Huh-7 cells and SK-HEP-1 were cultured in 24-well plates and treated with CIMs (containing 50 µg/mL IR780). After 0.5 h incubation at 37 °C, the treated 24-well plates were washed by PBS, and the cells were stained with DAPI, washed with PBS, and imaged by a confocal laser scanning microscope (CLSM; IX81, Olympus, Japan). To further investigate the targeting ability of particles, the phagocytosis of IR780 by cancer cells was measured at different time points. Again, 1×10^5^ Huh-7 cells were cultured in 24-well plates, and treated with IMs, TIMs and CIMs (containing 50 µg/mL IR780). After 1 h incubation at 37 °C, the treated 24-well plates were washed by PBS. To quantify IR780 uptake, the cells were harvested in 1 ml PBS and the IR780 amount in each group was measured by a UV-vis spectrometer. Phagocytosis at 2, 4, 8, 16, and 24 hours was measured by the same method. For the test on GPC3 overexpressed SK-HEP-1 cells, please see details in the Supplementary.

Biocompatibility was evaluated with the normal L02 liver cells. Cells were first cultured into a 24-well plate at a density of 1 × 10^5^ cells/well for 24 h. Various concentrations (0.50, 1.00, and 2.00 mg/mL) of CIMs were added into the cells, respectively, and maintained for 24 h at 37 °C in 5 % CO_2_. The viability of nanoparticle treated L02 cells was investigated using the Annexin V-FITC/PI Apoptosis Kit (Multi Sciences, China) following the standard instructions, and quantified by a flow cytometer. The biocompatibility of normal cells under illumination was also verified. Cells were first cultured into a 24-well plate at a density of 1 × 10^5^ cells/well for 24 h. Various concentrations (0.50, 1.00, and 2.00 mg/mL) of CIMs were added into the cells, respectively. After 24 h, the treated 24-well plates were washed by PBS and then exposed to 808 nm wavelength laser irradiation (0.6 W/cm^2^) for 5 min. The viability of L02 cells was investigated using the Annexin V-FITC/PI Apoptosis Kit (Multi Sciences, China) following the standard instructions, and quantified by a flow cytometer.

### *In vitro* phototherapy

The cytotoxicity of photothermal ablation for each group of nanoparticles was evaluated by Cell Counting Kit-8 (CCK-8, Dojindo, Japan) assay. Then, 8 × 10^3^ Huh-7 cells per well were seeded in 96-well plates, and cultured. After 24 h, the cells were treated with the following 6 groups (each group included 3 wells): (1) Control (PBS) (2) IMs (3) CIMs (4) IMs+NIR (5) TIMs+NIR (6) CIMs+NIR. The IR780 concentration was 50 µg/ml in groups 2, 3, 4, 5 and 6. After 2 h incubation, the culture media was refreshed to eliminate the suspended nanoparticles. Then, the cells in groups 4, 5 and 6 were irradiated by an 808 nm laser (0.6 W/cm^2^, 5 min). After treatment, CCK-8 solution was added as per instructions, and the cells were incubated for another 4 h. The cytotoxicity was calculated by dividing the optical density (OD) values of treated groups (T) by the OD values of the control (C) (T/C × 100 %).

Furthermore, Huh-7 cells were seeded in 96-well plates at a density of 5 × 10^3^ cells per well and incubated for 24 h. Then, the cells were incubated with the following 6 groups (each group included 3 wells): (1) Control (PBS) (2) IMs (3) CIMs (4) IMs+NIR (5) TIMs+NIR (6) CIMs+NIR. The IR780 concentration was 50 µg/ml in groups 2, 3, 4, 5 and 6. After 2 h incubation, the culture medium was refreshed to eliminate the suspended nanoparticles. Then, the cells in groups 4, 5 and 6 were irradiated by an 808 nm laser (0.6 W/cm^2^, 5 min). After treatment, cells were treated with LIVE/DEAD Viability/Cytotoxicity Kit according to the protocol and observed under a fluorescent microscope (IX81, Olympus, Japan).

The efficacy of phototherapy with various doses of CIMs in SK-HEP-1 and Huh7 cells under illumination was also tested. Cells were first cultured into a 6-well plate for 24 h. Various concentrations (0.50, 1.00, and 2.00 mg/mL) of CIMs were added into the cells, respectively. After 24 h, the treated 6-well plates were washed by PBS and then exposed to 808 nm wavelength laser irradiation (0.6 W/cm^2^) for 5 min. The viability of HCC cells was investigated using the Annexin V-FITC/PI Apoptosis Kit (Multi Sciences, China) and quantified by a flow cytometer.

### *In vivo* systemic toxicity, bio distribution and tumor imaging

All animal procedures in this study were performed according to the Institutional Animal Care and Use Committee at Wuhan University. For toxicity assessment, BALB/c-nu mice were intravenously injected with saline, IR780, IMs, and CIMs (4.5 mg/kg of IR780) twice every 3 days. At 19 days post-injection, the mice were killed, and the main organs including heart, liver, spleen, lung, and kidneys were collected and fixed into formalin for the paraffin embedding and sectioning. The organs were then stained with hematoxylin and eosin (H&E) to evaluate toxicity by histopathology.

For the subsequent studies, 5×10^6^ Huh-7 cells were subcutaneously injected into the flanks of 5-week old male BALB/c-nu mice. When tumors reached the size of ∼100 mm^3^, saline, IR780, IMs, and CIMs (1.5 mg/kg IR780) were intravenously (i.v.) injected into the mice (n = 5). At 24 h post-injection, fluorescent images were collected using an *in vivo* imaging system (Bruker Xtreme, USA) with 704 nm excitation wavelength.

For biodistribution assay, 150 µl free IR780, IMs and CIMs (1.5 mg/kg IR780) were intravenously injected when the tumor size reached about 100 mm^3^. The mice were killed at 12, 24 and 48 h post-injection to extract various tissues including the heart, liver, spleen, lung, kidney and tumor. To determine the contents of IR780, the tissues were further homogenized, and 2.5 mL methanol was added to extract IR780. The concentration of IR780 was then determined using fluorescence spectroscopy.

### Antitumor effect *in vivo*

To test the *in vivo* antitumor effect, 5×10^6^ Huh-7 cells were subcutaneously injected into the flanks of 5-week old male BALB/c-nu mice. The mice were divided randomly into 5 groups (each group included 5 mice): (1) Control (saline) (2) IMs (3) CIMs (4) IMs+NIR (5) CIMs+NIR. The IR780 dosage was 1.5 mg/kg in groups 2, 3, 4 and 5. Saline and various nanomaterials were injected intravenously. After 24 h post-injection, the tumor sites of mice in groups 4 and 5 were irradiated by an 808 nm laser (0.6W/cm^2^, 5 min). This procedure of administration and irradiation was conducted every 3 days for 19 days. Thermographic images were taken by an infrared thermal camera (A150-15-M, Irtech Ltd.), and the photothermal temperatures at various times were recorded. Animal weight and tumor volume were recorded every 3 d until the end of the experiment. Tumor volume (V) was calculated by the formula: V = (Length × Width2)/2. On the 19th day of the administration period, mice were euthanized by asphyxiation and the tumors were then extracted for terminal deoxynucleotidyl transferase dUTP nick end labeling (TUNEL) staining.

### Statistical analyses

The data were presented as mean values ± SD and each value represented the mean of at least three repetitive experiments in each group. Non-parametric test was performed using GraphPad Prism 7.0 to assess the significance of the difference between two groups, *p < 0.05 and **p < 0.01.

## Results and Discussion

### Construction of CAR-T cells and their ability to target GPC3 positive HCC cells

**Figure 2:**
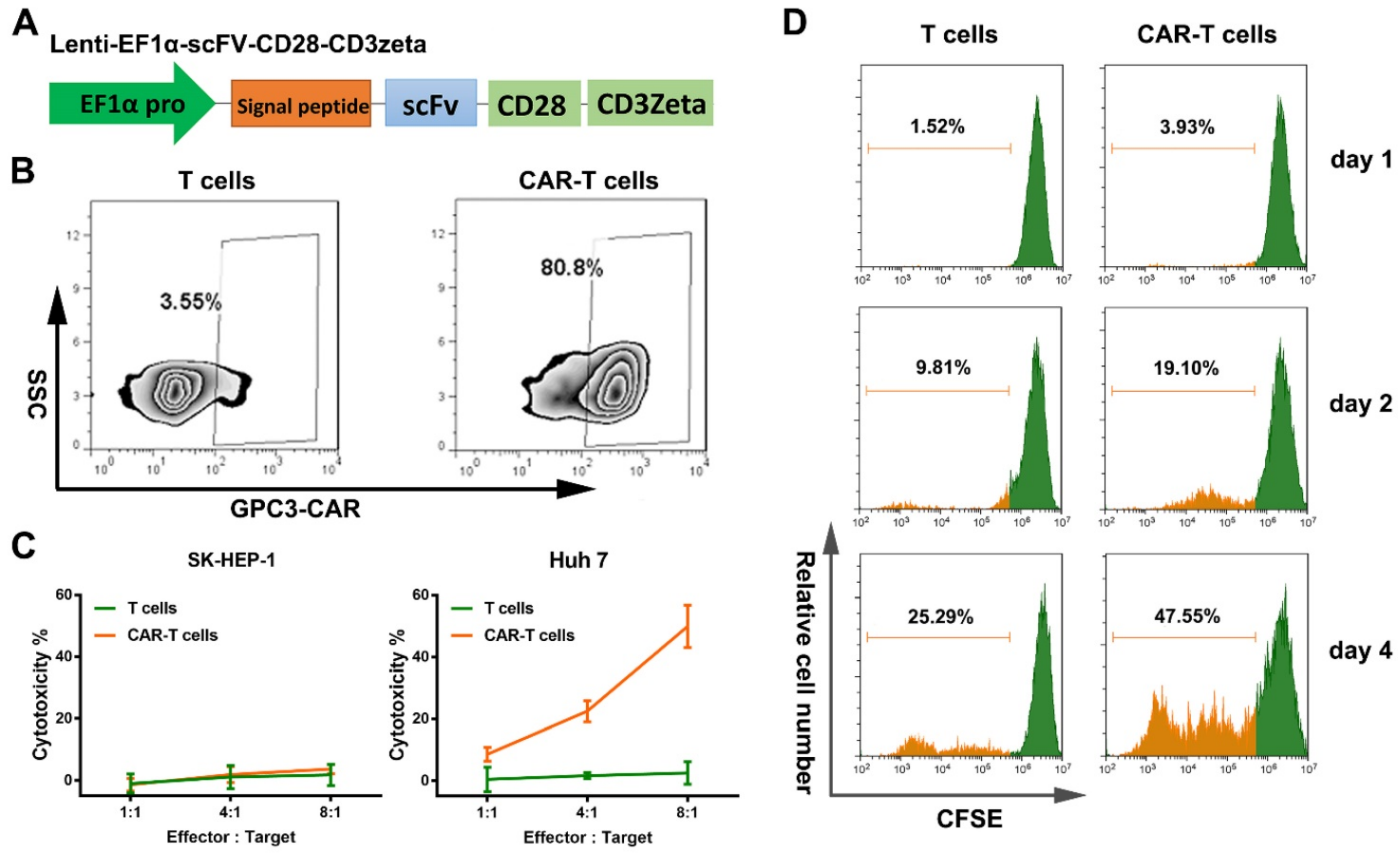
** Construction and verification of GPC-3 targeted CAR-T cells. A.** Schematic representation of a lentiviral CAR vector. The GPC3 targeted ScFv derived from GC33 monoclonal antibody was linked to human CD8α hinge and CD28 transmembrane domain. Control T cells were transfected by lentivirus with empty vector. **B.** The efficiency of transduction was measured using flow cytometry by detecting the GC33 ScFv expression on the cell surface of T cells and CAR-T cells, respectively. **C.** The cytotoxicity of T cells and GPC3-CAR-T cells was tested. Either T cells or CAR-T cells were co-incubated with Huh-7 and SK-HEP-1 at varying effector:target ratios for 18 hours. Cell lysis was then measured through LDH cytotoxicity assays. **D.** Cell proliferation of T cells and CAR-T cells was detected at day 0, day 2, and day 4. The figures are representative of three independent experiments. Each data point reflects the mean± SD of triplicates.

Blood derived T cells were separated and activated with CD3 and CD28 for 48 h, and transduced with GPC3-CAR lentivirus. At day 14 post-transduction, flow cytometry results showed transduction efficiencies of approximately 81-86 % (Figure 2B). The T cell population after the construction of CAR-T cells which was similar between T cells and CAR-T cells (Figure S1A). Next, LDH assays were performed to assess the cytotoxicity of GPC3-CAR-T cells. The T cells or CAR-T cells were co-cultured with GPC3 over expressing HCC cells (Huh-7 cells) or GPC3 under expressing HCC cells (SK-HEP-1 cells) (shown in Figure S1B and C) at different E: T ratios (1:1, 1:4, 1:8). In this process it was apparent that GPC3-CAR-T cells were much more cytotoxic to Huh-7 cells than SK-HEP-1 cells (Figure 2C), and the T cells did not show significant cytotoxicity to either HCC cell groups. The CAR-T cells and T cells proliferation at different time point, day 1, day2 and day 4 after the treatment of CFSE reagent (Figure 2D). The proliferation rate of CAR-T cells was obviously enhanced compared with T cells. The high proliferation ability of CAR-T cells was caused by the intracellular costimulatory molecules, CD28 and CD3zeta which were the components of CAR structure. It proved the successful construction of CAR-T cells. These findings indicated that the genetically modified CAR-T cells could precisely target GPC3 positive HCC cells in contrast to normal T cells.

### Preparation and Characterization of CIMs

**Figure 3:**
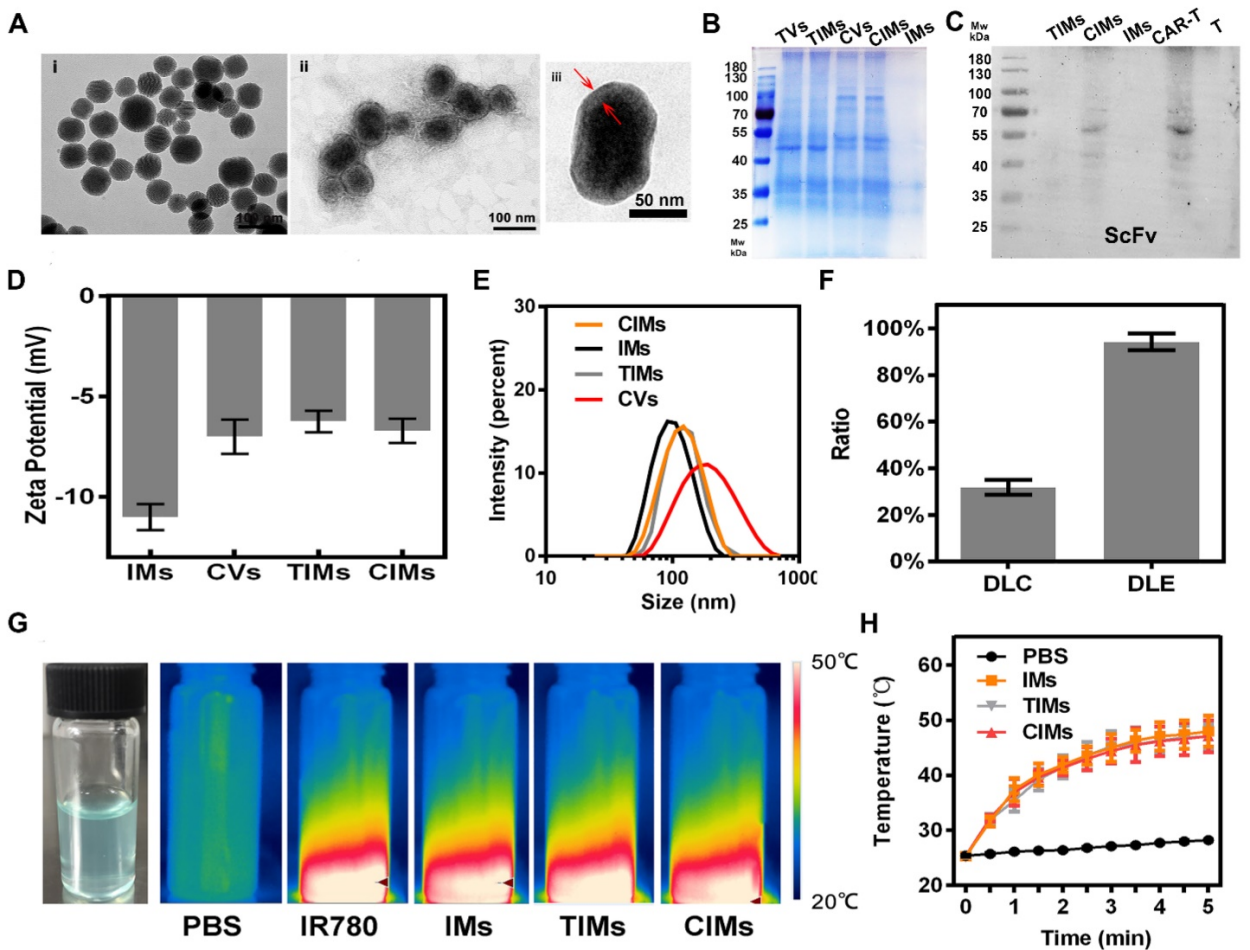
** Preparation and characterization of CIMs. A.** TEM images of i) IMs and ii) CIMs and iii) CIMs with high magnification. **B.** SDS-PAGE protein identification images of TVs, CVs, TIMs, CIMs, and IMs. **C.** GC33 ScFv on CAR-T cell membrane and CIMs was detected by western blot. **D.** Hydrodynamic zeta potentials. **E.** Diameters of TVs, CVs, CIMs, and IMs. Error bars: standard deviations (n=3). **F.** IR780 loading content (DLC) and IR780 loading efficiency (DLE) of CIMs. **G.** Physical image of CIMs and infrared thermalgraphic images of PBS, IR780, CIMs, and IMs at 5 min of NIR laser irradiation. **H.** Temperature elevation curves of PBS, IMs, and CIMs at 5 min of NIR laser irradiation. Error bars: standard deviations (n=3).

Transmission electron microscope (TEM) was used to observe the morphology of IMs and CIMs. As seen from the TEM images in Figure 3A and Figure S2A, the size of CIMs (about 110 nm) was larger than that of IMs (about 95 nm), and the thickness of the CAR-T layer was almost 5 nm. Threshold levels of protein content in various nanoparticles were determined by SDS-PAGE and western blot, as illustrated in Figure 3B and 3C. The results indicated the difference in protein content between TV and CV. More importantly, similar protein content was found in CV and CIMs, while almost zero protein content was in the band of IMs. These results indicate the successful translocation of the CAR-T membrane proteins from cells to IMs. As shown in Figure 3D, the mean zeta potential of CVs and CIMs were -7 mV and -6.7 mV, which were higher than that of IMs (-11 mV). The negative charge of CAR-T vesicle surface caused the change in zeta potential. As seen from Figure 3E, the average hydrodynamic diameters of IMs (particle size distribution from 68 nm to 142 nm, PDI=0.135), TIMs (particle size distribution from 79 nm to 142 nm, PDI=0.262), CIMs (particle size distribution from 91 nm to 164 nm, PDI=0.288) and CVs (particle size distribution from 106 nm to 345 nm, PDI=0.326) were 105 nm, 122nm, 135 nm and 201 nm, respectively. The hydrodynamic diameter and zeta-potential results show that CAR-T and T vesicles successfully coated the nanoparticles. The IR780 loading content (DLC) and loading efficiency (DLE) in the experiment were 31.8±3.2 % and 94.2±3.6 %, respectively, (Figure 3F) indicating the high drug loading capacity of CIMs. As shown in Figure S2B, IR780 and IMs had strong absorption at 780nm. On the other hand, TIMs and CIMs showed obvious red shift and the maximum absorption peak was around 800 nm, which was possibly caused by membrane wrapping. The results of *in vitro* drug release experiments showed that the release rate in IMs without membrane coating was very fast (Figure S2C), while the release rate of IR780 in TIMs and CIMs was significantly slowed down. This indicates that the membrane coating was beneficial to improve the problem of sudden or instantaneous release. During the experiments, IR780 was found to degrade rapidly under light (Figure S2D and E), so drug release experiments under radiation were not designed. The above results were consistent with previously reported research 28. To verify the right orientation of membrane proteins after the cell membrane coating on IMs, CD3 was quantified by flow cytometry on CIMs surface and comparing with that on CAR-T cells membranes with an equal amount of membrane proteins. No significance was detected in CD3 level between CIMs surface and CAR-T cells membranes (Figure S2F). As membrane coating on CIMs was derived from the CAR-T cell membrane, this result indicated the right-side-out protein which meant the right ScFv extending from the surface of CIMs. In addition, thermal imaging showed the excellent photothermal conversion of IR780, as illustrated in Figure 3G-H. The IMs and CIMs exhibited similar temperatures after being exposed to NIR irradiation for 5 min, showing that the cell membrane coating had insignificant influence on the photothermal ability of IR780. The temperature of nanoparticle-free phosphate buffer solution (PBS) did not increase. Then photothermal conversion efficiency of CIMs is 22.2% (Figure S2G and H). The superior photothermal conversion of IR780 enables it to destroy cancer cells via hyperthermia (42-47 °C) 34.

### Evaluating of the targeting ability and biocompatibility of CIMs

**Figure 4:**
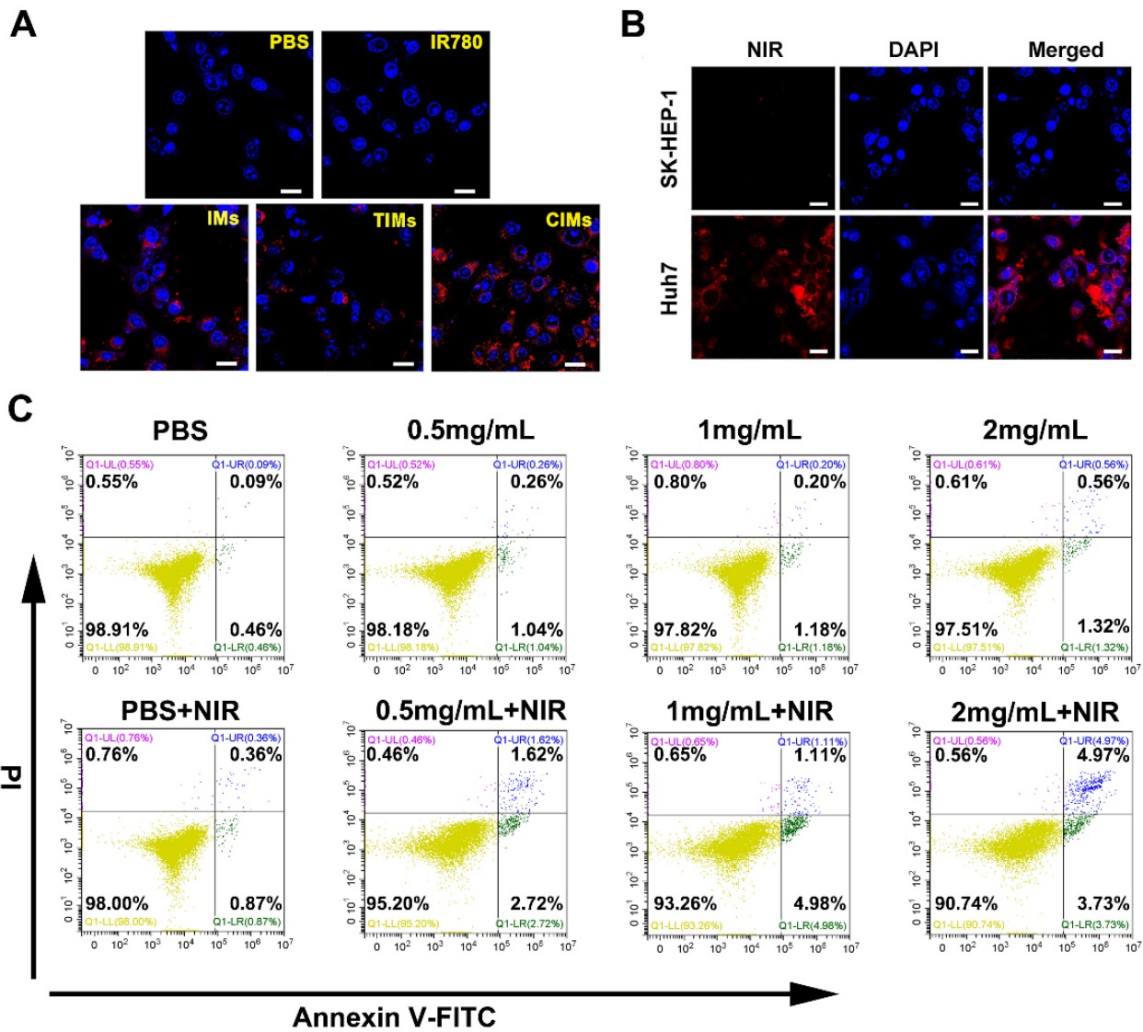
***In vitro* targeting capability and biocompatibility evaluation. A.** CLSM photos of Huh-7 cells after incubation with various nanoparticles. Cells cultured without the addition of any nanoparticles were used as the control. Scale bar: 20 μm. **B.** CLSM images of Huh-7 cancer cells and SK-HEP-1 cells after incubation with CIMs. Scale bar: 20 μm. **C.** Flow cytometry analysis of normal L02 cells after treating with CIMs at different concentrations. Positive PI and Annexin V-FITC cells were defined as late apoptosis/necrotic cells.

The Huh-7 cells were cultured with the same quantity of IMs and CIMs nanoparticles, and IR780 fluorescent signal (700-800 nm) was detected by CLSM at an excitation wavelength of 633 nm. The cells treated with CIMs exhibited bright red fluorescence, and the number of CIMs bound to cells was dramatically increased compared with other nanoparticles (Figure 4A and B). These results demonstrate the ability of CIMs to target cancer cells *in vitro*. The mean IR780 fluorescence intensity in each group was further determined by flow cytometry (Figure S2I). The results showed that the fluorescence intensity of the CIMs group was 10 and 100 times higher than that of the IMs and IR780 groups, respectively. It was also much higher than that of TIMs. This result indicates that more nanomaterials targeted the tumor cells in the CIMs group, and that CIMs have more robust targeting ability than IMs and IR780. Previous studies have found that IR780 was retained in the mitochondria of cancer cells 35. Thus, it is not unexpected to see higher tumor targeting in IR780 and IMs groups when compared to the control. It is worth noting that T cell membrane has a variety of proteins on its surface to detect inflammation and diseased tissues 36, 37, while CAR-T cells are manufactured with tumor targeting ability compared to their native counterparts.

Therefore, by coating the novel nanomaterials with CAR-T membranes, their targeting ability was inherited by the nanomaterials. Then, the nanoparticle uptake by Huh-7 cells was quantitatively analyzed with various incubation time periods at the initial IR780 concentration of 50 μg/ml. As shown in Figure S2J, IR780 content in cells in the CIMs group increased the most rapidly, which was also due to its excellent tumor cell targeting ability.

Biocompatibility of nanomaterials is a necessary precondition for biomedical applications 38. To investigate the biocompatibility of the CIMs, flow cell death assays were performed on normal liver L02 cells. Figure 4C shows the viability of cells cultured with CIMs at various concentrations in comparison with untreated controls. Even at relatively high concentrations (2 mg/mL) of CIMs, the cell survival rate is over 90%, demonstrating good biocompatibility and safety towards normal cells. In addition, due to the weak targeting ability of CIMs for normal cells, the apoptosis rate of L02 cells was also very low under NIR treatment.

### *In vitro* phototherapy

**Figure 5:**
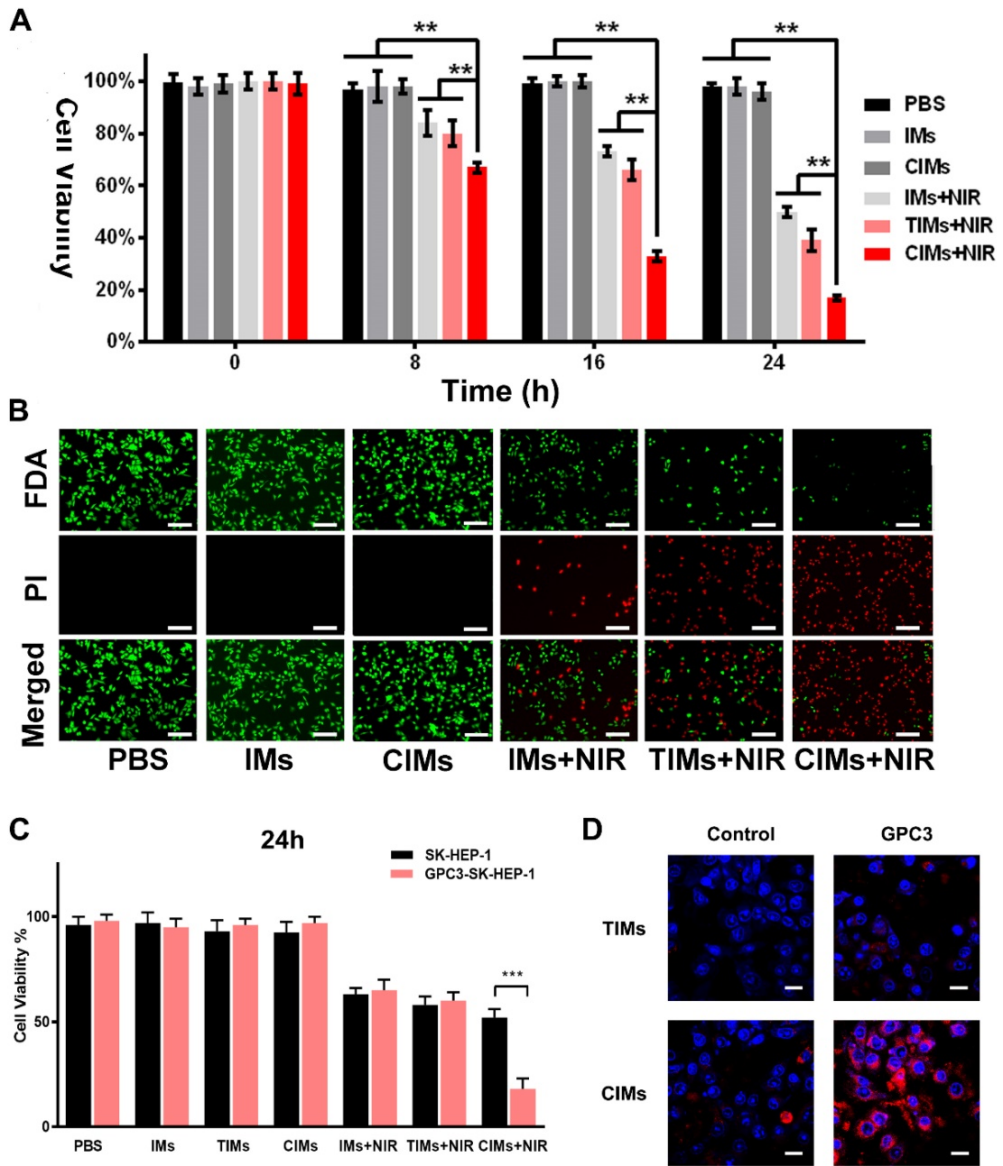
***In vitro* phototherapy. A.** *In vitro* cytotoxicity of PBS, IMs, CIMs, IMs with NIR laser irradiation, TIMs with NIR laser irradiation and CIMs with NIR laser irradiation at different time points. The data are shown as mean±SD (n = 3). **B.** Photothermal cytotoxicity images of the nanoparticles on Huh-7 cells. Live cells were stained by fluorescein diacetate (FDA) (green), and the dead cells were stained by PI (red). Scale bar = 100 μm. **C.** *In vitro* cytotoxicity of PBS, IMs, CIMs, IMs with NIR laser irradiation, TIMs with NIR laser irradiation and CIMs with NIR laser irradiation at 24h after treatment. The data are shown as mean±SD (n = 3). **D.** CLSM photos of GPC3 overexpressed SK-HEP-1 cells and control after incubation with TIMs and CIMs. Scale bar: 20 μm. *, *P*<0.05; **, *P*<0.01.

*In vitro* photothermal therapy was carried out on Huh-7 cells. The results of CCK-8 (Figure 5A) showed a high survival rate for tumor cells treated with different nanoparticles. In the IMs + NIR, TIMs+NIR and CIMs + NIR groups, the survival rate of cells was rapidly reduced over time to 50 %, 39 % and 17% at 24 hours, respectively. Similar results also be proved by flow cytometry assays in Figure S3A. This finding indicates the high hyperthermic cytotoxicity of IR780. In addition, it shows that CIMs were more effective than IMs and TIMs, and that the CAR-T membrane provided better targeting towards tumor cells and antitumor effects. A live/dead cell staining kit was also used to directly observe cell death. As illustrated in Figure 5B, PBS and IMs treated cells showed bright FDA green fluorescence, indicating high survival rate. IMs + NIR, TIMs + NIR and CIMs + NIR treatments resulted in low survival rates, with CIMs + NIR being more effective. This result was consistent with the CCK-8 result. Besides, the specific targeting ability and NIR-induced tumor killing ability of CIMs were confirmed on GPC3 overexpressed SK-HEP-1 cells (Figure 5C-D). As the upregulation of GPC3 protein on SK-HEP-1 cells which was GPC3 negative (Figure S2K), the targeting ability of CIMs was better performed. While, no significance was observed on TIMs. Overall, the results demonstrate that CIMs have superior antitumor effects.

### *In vivo* antitumor effect

**Figure 6:**
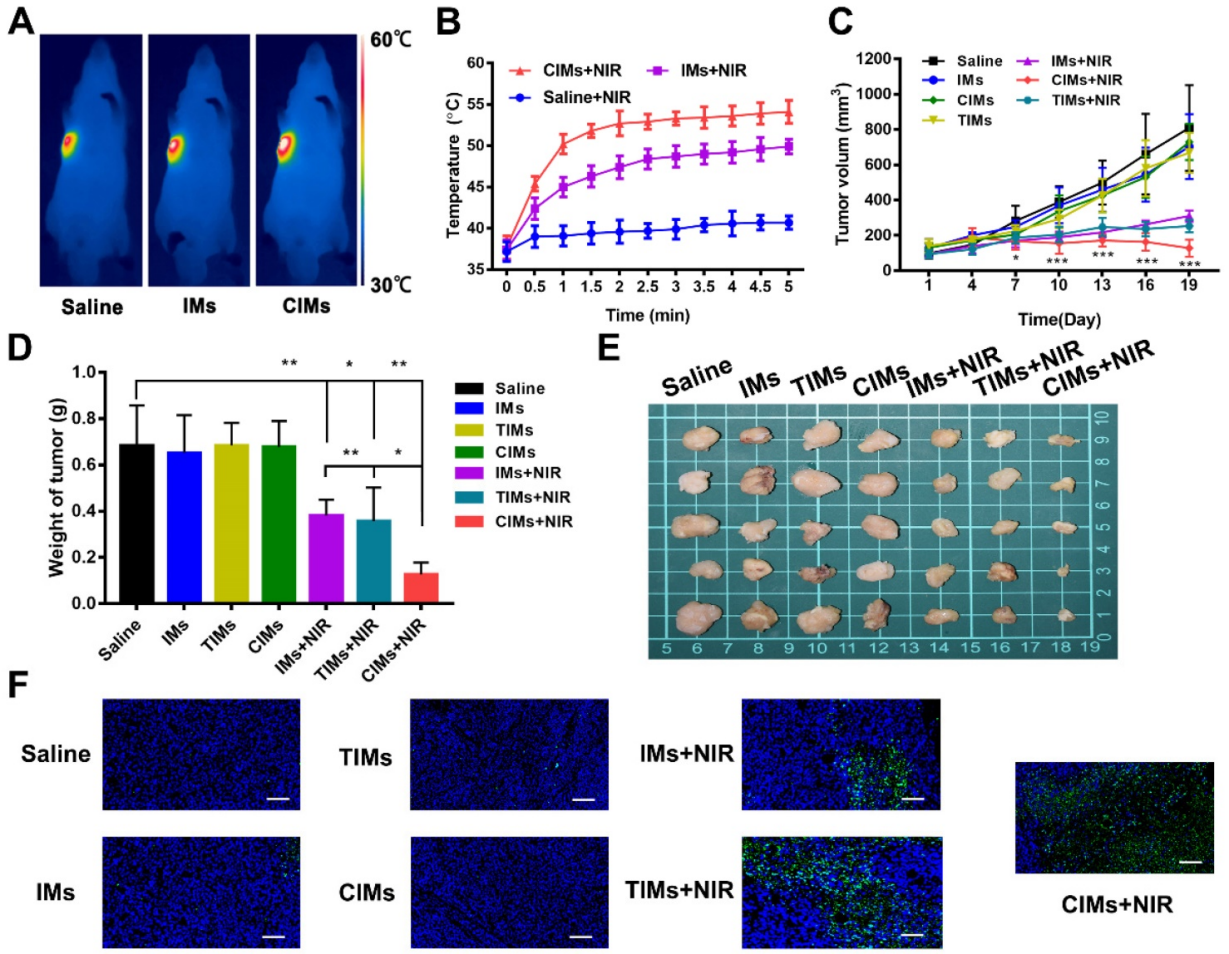
***In vivo* anti-tumor effects. A.** Infrared thermographic images of Huh-7 tumor-bearing nude mice after NIR irradiation. **B.** Temperature increase behaviors of the tumor tissues in the mice after receiving intravenous injection with saline, IMs, and CIMs with NIR irradiation. The data is shown as mean±SD (n = 3). **C.** Tumor growth profiles. **D.** Weight of tumors of the dead mice at day 19. The data is shown as mean±SD (n = 5). **E.** Ex vivo images. **F.** TUNEL staining of the tumor slices. Scale bar = 100 μm. *, *P*<0.05; **, *P*<0.01; ***, *P*<0.001.

The antitumor ability of CIMs was studied in mice under a subcutaneous liver cancer model. As shown in Figure 6A, when the tumor was exposed to laser irradiation for 5 min, IMs and CIMs groups experienced a significant temperature rise of 15 °C, with the final temperature reaching more than 50 °C. Such temperatures are sufficient for hyperthermic killing of tumor cells. In the saline group, the temperature rose to only 40 °C, which is not high enough to kill cells. The temperature was then monitored at regular intervals over a period of 5 min, and a temperature curve was generated (Figure 6B). The temperatures of CIMs and IMs groups rose quickly in the first 2 min, indicating good thermal conversion efficiency of IR780. In addition, CIMs resulted in higher temperatures than IMs, confirming that CIMs were specifically enriched in tumor tissues, resulting in an enhanced photothermal effect. Changes in tumor volume and weight were also observed (Figure 6C and E). In the saline and IMs groups, the tumor tissue grew rapidly. When IMs or TIMs were injected and the tumor was irradiated, the tumor volume was reduced by 50 %, indicating the ability of IMs and TIMs to induce tumor cell death via a photothermal effect. In the CIMs+NIR group, the tumor volume was further reduced compared with IMs+NIR and TIMs+NIR, demonstrating the enhanced localization from the CAR-T membrane coating. While, no significance was observed in tumor volume between IMs+NIR and TIMs+NIR. There was also no significant reduction in body weight for each group of mice, indicating that the material had no serious systemic toxicity (Figure S3B). Using TUNEL staining, the tumor inhibition was mechanistically associated with tumor death as illustrated in Figure 6F. No significant fluorescence or cell death was observed in the saline and IMs groups, while significant cell death was observed in CIMs+NIR and IMs+NIR groups, with CIMs+NIR showing maximal effect. Through the CLSM photos of distribution of IMs, TIMs and CIMs in tumor, the red fluorescence signal for IR780 was stronger for CIMs relative to IMs or TIMs (Figure S3C), indicating enhanced tumor targeting ability of CIMs *in vivo*.

### *In vivo* tumor imaging, biodistribution, and systemic toxicity assessment

**Figure 7:**
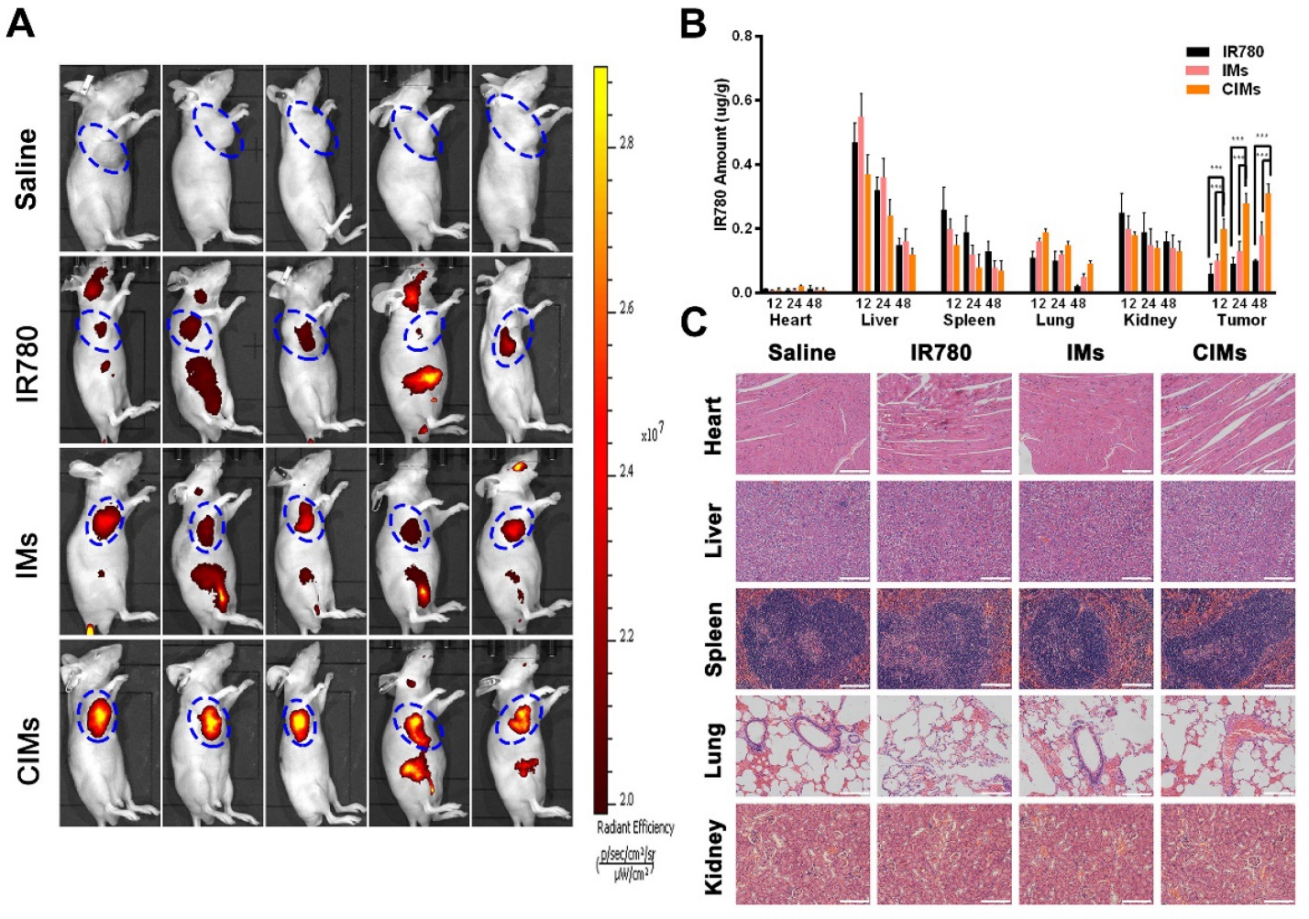
***In vivo* bio-distribution and systemic toxicity. A.** *In vivo* imaging of tumor-bearing mice at 24 h post-injection treated with IR780, IMs, and CIMs at a dose of 0.2 mg/kg IR780, respectively. The blue circles indicated the location of tumors. **B.** Ex vivo amounts of IR780 from IMs, CIMs, and free IR780 in the tumors of the tumor-bearing mice at the dose of 1.5 mg/kg IR780 at 48 h post-injection, respectively. The data is shown as mean ± SD (n = 5). Scale bar = 100 μm. **C.** Histopathologic examination of the tissues including heart, liver, spleen, lung, and kidney from nude mice after intravenous administration of saline, IR780, IMs, and CIMs for 19 d. *, *P*<0.05; **, *P*<0.01; ***, *P*<0.001.

Near-infrared fluorescence tumor imaging was carried out on the Huh-7 subcutaneous hepatocellular carcinoma model. As shown in Figure 7A, at 24 h post-injection, the CIMs group displayed the strongest fluorescence signal at the tumor site, while IR780 and IMs groups exhibited weaker fluorescence, indicating better targeting by CIMs. This finding also suggests that CIMs is an excellent material for near-infrared tumor imaging. The levels of IR780 in each organ and tumor tissue at 12-, 24- and 48 h post-injection were also analyzed, as seen in Figure 7B. As expected, the content of IR780 in tumor tissues for the CIMs group was much higher than that of the IMs and IR780 groups, which was consistent with the live imaging results and confirmed the superior targeting by CIMs. Normal nude mice were also used to study the side effects of the nanoparticles on internal organs (Figure 7C). After H&E staining of the main organs (heart, liver, spleen, lung, and kidney), no obvious organ damage was observed, indicating that CIMs have excellent biocompatibility *in vivo*, and no short-term side effects.

## Discussion

The CAR, which is based on the T cell receptor, is an artificially modified fusion protein which contains an extracellular antigen recognition domain fused to various intracellular signaling domains. In most cases, the extracellular domain is the single-chain variable region (ScFv) of monoclonal antibody heavy and light chains which can directly recognize the TAA on tumor cell surface without the presentation from MHC molecules 39. The intracellular domains include CD3ζ, CD28, 4-1BB, or OX40, and are devised to enhance the activation of T cells 39. When CAR-T cells identify tumor cells expressing the target TAA on a cell surface, T cell activation, proliferation, cytokine secretion, and cytotoxicity toward TAA-expressing tumor cells are triggered. The field of CD19-targeting CAR-T cell therapy has experienced dramatic progress in hematological cancers such as acute lymphoblastic leukemia 40.

However, CAR T cells face a unique set of challenges in the field of solid tumors 41. To achieve a favorable clinical outcome, CAR T cells have to overcome a series of increasingly arduous barriers. First, the specific antigens which are expressed should be able to clearly demarcate the tumor from normal tissue. Then, CAR-T cells should be able to find and penetrate the desmoplastic stroma that surrounds the tumor. Once the cells are within the tumor, they must expand, persist and mediate cytotoxicity in a hostile milieu largely composed of immunosuppressive modulators 41, 42. While a seemingly herculean task, all the above requirements can potentially be surmounted effectively through intrinsic or extrinsic modifications of CAR-T cells.

The application of nanotechnology in medicine is providing significant opportunities and new perspectives for novel and effective treatments in many diseases. Nanomedicine can be defined as the design and development of therapeutics and/or diagnostic agents in the nanoscale range (with diameters ranging from 1 nm to 1,000 nm), with the possibility to move within biological systems, and transport and deliver a variety of biomedical entities for the treatment, prevention, and diagnosis of many diseases 43. Due to their unique characteristics, including large surface area, structural properties, and long circulation time in blood compared with small molecules, nanoparticles have emerged as attractive candidates for optimized therapy through personalized medicine 44, 45.

Attempts have been performed on the combination of nanomedicine and cell therapy, especially T cell therapy. Rachel A Burga et al. generated a biohybrid nano-immunotherapy using antigen-specific T cells as vehicles for Prussian blue nanoparticles (PBNPs). They conjugated PBNPs onto primary EBV antigen-specific cytotoxic T lymphocytes (CTL) and the conjugation process maintained the individual functions of both the PBNPs and the CTL. These studies suggest the potential use of our biohybrid CTL:PBNPs as a therapeutic for the treatment of cancer 46. Matthias T. Stephan et al. used the method of conjugation of adjuvant drug-loaded nanoparticles to the surfaces of therapeutic cells to provide sustained pseudo-autocrine stimulation to donor cells. Dramatic enhancements in tumor elimination in a model of adoptive T-cell therapy for cancer were detected and increased the *in vivo* repopulation rate of hematopoietic stem cell grafts, using very low doses of adjuvant drugs that were ineffective when given systemically 47. These works could be proof of concept study demonstrating the feasibility of the application of nanoparticles on T cell therapy. The synthetic polymer poly (ethylene glycol) (PEG) which they used could create a hydration layer, while also providing steric stabilization lead to a stealthy nanoparticle surface that interacts less with its environment, enabling significantly enhanced blood circulation. A wide range of ligands, including antibodies, aptamers, peptides, and small molecules, to add targeting functionality. It has been proven that PEG was effective at minimizing nonspecific interactions in complex media, however, increasing reports about immune response against the synthetic polymer and the antibodies against PEG can potentially impact performance over multiple administrations, let alone the difficulties for large-scale manufacturing of bottom-up targeting ligand conjugation strategies 48, 49.

Cell membrane-coated nanoparticles, characterized by a synthetic nanoparticle core cloaked by a layer of natural cell membrane, inherently mimic the properties of the source cells from which their membrane is derived, and thus exhibit a wide range of functions such as long circulation and disease-relevant targeting 50. Different types of membrane coatings with special features are currently employed, such as red blood cells (RBCs) membranes, cancer cell membranes and platelet membrane membranes 51-53. Their advantages for specific applications are covered in depth, which uniquely benefit from the presence of biological membranes. A layer-by-layer hybrid nanoparticles has been designed, which enabled the release of IR-780 dye for NIR-induced photothermal and photodynamic effects, and the release of imatinib-loaded glucocorticoid-induced TNF receptor family-related protein/poly(lactic-co-glycolic acid) (GITR-PLGA) nanoparticles to initiate antitumor immunotherapy. The photothermal and photodynamic effects caused by IR-780 under NIR exposure resulted in direct tumor apoptosis/necrosis and the production of tumor-associated antigen, promoted dendritic cell maturation, and enhanced the presentation of tumor-associated antigen to T cells, while the imatinib-loaded GITR-PLGA cores reduced the suppressive function of Treg cells, and consequently activated effective CD8^+^ T cells towards tumors 54. Lianru Zhang et.al reported a biomimetic delivery platform based on human cytotoxic T-lymphocyte membranes camouflaged poly-lactic-co-glycolic acid nanoparticles. This drug-delivery platform retained both the long circulation time and tumor site accumulation ability of human cytotoxic T lymphocytes 55. This indicated, coating with CTL cell membranes could gift tumor targeting ability to nanoparticles. While, as we all known, the gene-engineered CAR-T cells obtain enhanced specific tumor targeting ability in comparison with T cells. Therefore, it inspired us to use CAR T membrane coated nanoparticles for targeted tumor therapy.

In this study, the outstanding targeting ability of CAR-T cells was recruited along with the photothermal ability and advantage in drug delivery of nanoparticles by cell membrane coating method. By coating with the membranes of GPC3-specific CAR-T cells, the novel nanoparticles, CIMs, could inherent the targeting ability and be bestowed with a long circulation. First, we designed and constructed the GPC3 specific CAR-T cells by lentivirus transfection. Through the verification of the enhanced T cell proliferation and cytotoxicity towards GPC3^+^ HCC cells, the unique characteristics of CAR-T cells were confirmed which indicated the successful construction of GPC3 specific CAR-T cells (**Figure 2**). Next, membranes of CAR-T cells and T cells were isolated and coated onto IR780 loaded meso-porous silica nanoparticles to construct different novel nanoparticles (**Figure 3**), which were then thoroughly characterized, like zeta potential and hydrodynamic diameters. The larger size of CIMs was observed from TEM. The right membrane proteins content and orientation were also confirmed which indicated the right ScFv extending from the surface of CIMs and further guarantee of tumor targeting ability inherited from CAR-T cells. The photothermal potential of these novel nanoparticles was also confirmed. IR780 contained CIMs had strong absorption at around 800nm. Coating with the membranes could slow down the release rate of IR780 and stabilize the CIMs but didn't impair the photothermal conversion of IR780. Furthermore, the results of *in vitro* and in HCC tumor bearing mouse models confirmed that CIMs inherited the excellent tumor targeting ability from GPC3 specific CAR-T cells and possessed significant photothermal antitumor abilities. The well biocompatibility of CIMs was also evaluated. Without NIR, these novel nanoparticles barely affected the cell viability. Once treated with 808 nm NIR, the photothermal ability of each nanoparticle was induced which led to the death of HCC cells. Besides, under NIR treatment, CIMs displayed excellent tumor imaging abilities *in vivo*.

In this study, we recruited the outstanding targeting ability of CAR-T cells along with the photothermal ability and advantage in drug delivery of nanoparticles by cell membrane coating method. By coating with the membranes of CAR-T cells, the novel nanoparticles can inherent the targeting ability and be bestowed with a long circulation. The nano-sized nanoparticles could be easier home and penetrate the desmoplastic stroma that surrounds the tumor. However, before the practical clinical use, these novel nano-CAR-T therapies still need more tests to verify its efficacy and safety.

## Conclusion

In this study, GPC3-targeting CAR-T cells were constructed, and their membranes were extracted and coated onto IR780 loaded meso-porous silica nanoparticles that can produce heat and fluorescence under laser irradiation. *In vitro* and *in vivo* experiments were then conducted to illustrate the enhanced tumor targeting ability, minimal systematic toxicity, and excellent photothermal response of the novel photothermal therapeutic agent. These findings indicate that novel CAR-T cell membrane-coated nanoparticles can be developed for cancer therapy in the future.
