# Supplementary material for: Coating biomimetic nanoparticles with chimeric antigen receptor T cell-membrane provides high specificity for hepatocellular carcinoma photothermal therapy treatment
Source: Theranostics. 2020 Jan 1;10(3):1281–95. doi: 10.7150/thno.40291 (PMC6956810; doi:10.7150/thno.40291)
Supplement: Supplementary file 1 — Supplementary materials and methods, figures. [file thnov10p1281s1.pdf]

Supplementary

Materials and Methods

In vitro targeting ability of CIMs on GPC3 overexpressed HCC cells

After being seeded into the 6-well plates overnight, SK-HEP-1 cells were transiently transfected with GPC3 plasmids (Addgene, USA) by Lipofectamine 3000 transfection reagent (Invitrogen, CA). The overexpression level of GPC3 protein was detected by western blot after 48-hour transfection. Then, targeting ability and cytotoxicity of photothermal ablation of each nanoparticles on GPC3 overexpressed SK-HEP-1 were tested as mentioned before.

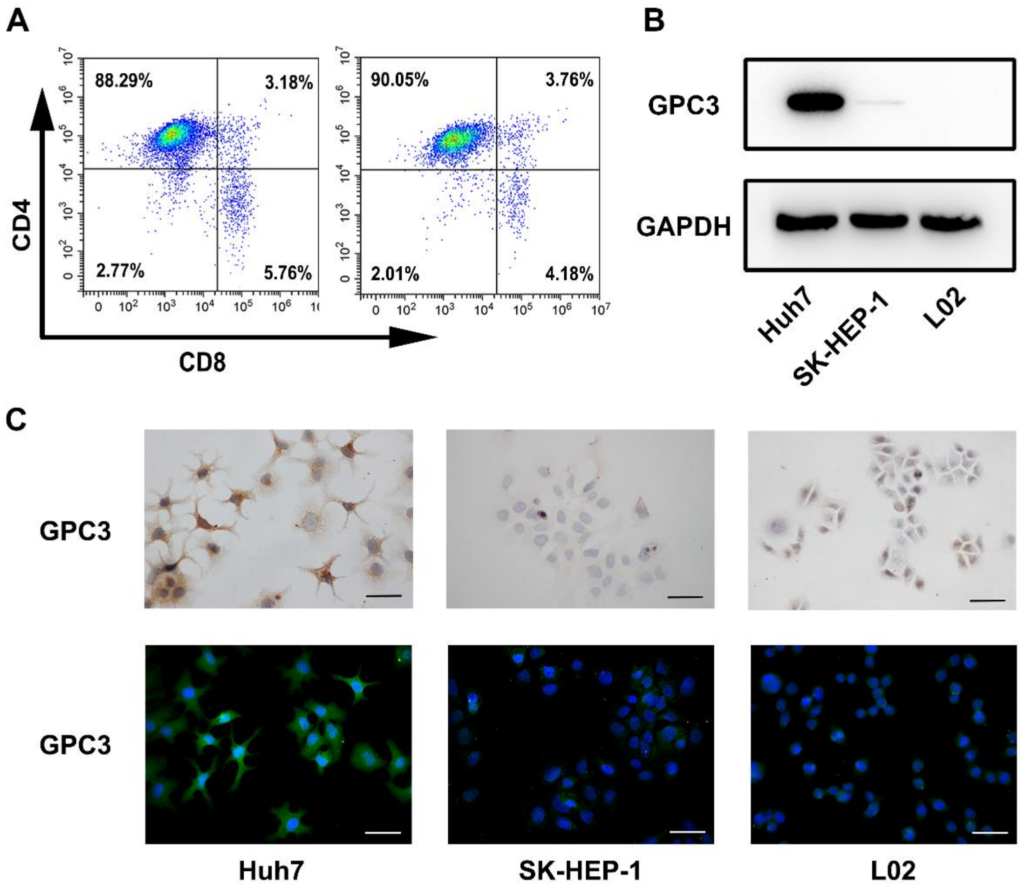

Figure S1. Detection of GPC3 in different cell lines and the construction of membrane coated nanoparticles. A. Characterization of the T cell population identified through flow cytometry analysis. $CD4^{+}/CD8^{+}$ T cell population was similar after the CAR lentivirus infection. **B.** The western blot results of GPC3 expression in Huh7, SK-HEP-1 and L02 cell lines. **C.** immunohistochemistry (upper) and immunofluorescence (lower) of GPC3 protein in Huh7, SK-HEP-1 and L02 cells indicated that the GPC3 protein was located on the cell membranes. In addition, GPC3 was relatively overexpressed in Huh7 cells.

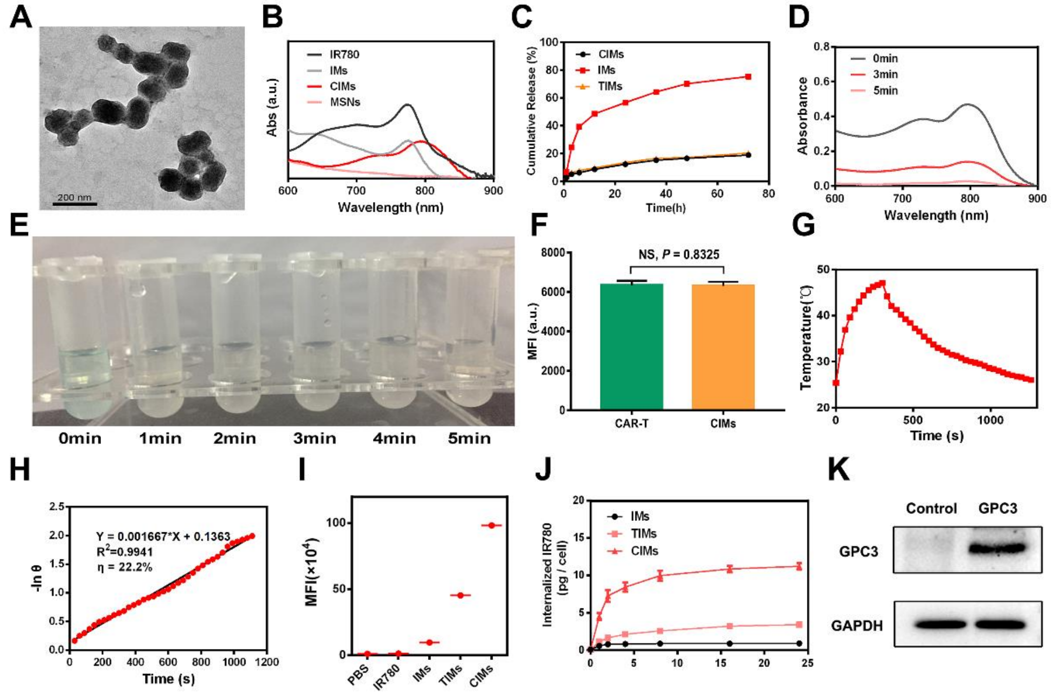

Figure S2. A. TEM image of TIMs. **B.** UV–vis absorption spectra of IR780, IMs, MSNs, TIMs and CIMs. **C.** In vitro IR780 release behavior from IMs, TIMs and CIMs in PBS at 37°C. **D.** Changes in the UV-Vis absorbance of CIMs with increase in irradiation time. **E.** Changes in the color of CIMs dispersions with increase in irradiation time. The initial concentration of IR780 was 20 μg/ml. **F.** Comparison of fluorescence intensity measured in CAR-T cells (~1 × 10^6 cells) and neutrophil-NPs (100μL of suspensions, 0.15 mg/ml protein content) stained with APC anti-Human CD3 antibody. NS, no significance. **G. In vitro photothermal ability of CIMs nanoparticles.** The photothermal response of CIMs PBS solution (808 nm, 0.6W/cm^2) with a NIR laser (808 nm, 0.6W/cm^2), and then the laser was shut off (containing 50 μg/mL IR780). **H.** Linear time data versus −lnθ obtained from the cooling period of **Figure S2G**. **I. Mean**

fluorescence intensity (MFI) of IR780 from Figure 4A. Flow cytometric analysis for mean fluorescent intensity (n = 10 000 cells) in Huh-7 cells incubated with different nanoparticles for 30 min. **J.** Quantitative analysis of the nanoparticle uptake by Huh-7 cells with various incubation time periods at the concentration of 50μg/ml IR780. **K.** GPC3 overexpression on SK-HEP-1 cells.

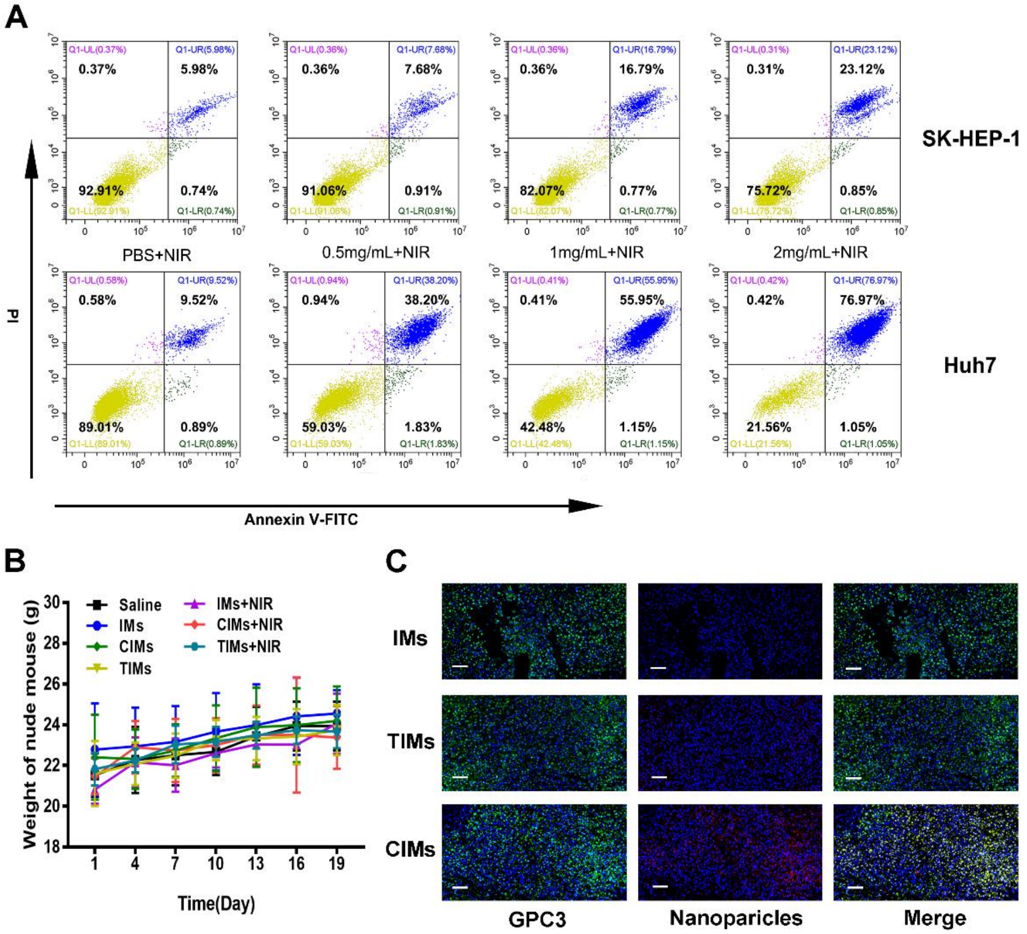

Figure S3. A. Flow cytometry analysis of SK-HEP-1 and Huh7 cells after treating with various doses of CIMs and laser irradiation. Positive PI and Annexin V-FITC cells were defined as late apoptosis/necrotic cells. **B.** Weight changes of mice. **C.** CLSM photos of

distribution of IMs, TIMs and CIMs in tumor. DAPI stained for the nuclear, green marked the GPC3 protein and red indicated the location of each IR-780 containing nanoparticles. Scale bar = 50 μm.
